# Botulinum Neurotoxin A4 Has a 1000-Fold Reduced Potency Due to Three Single Amino Acid Alterations in the Protein Receptor Binding Domain

**DOI:** 10.3390/ijms24065690

**Published:** 2023-03-16

**Authors:** William H. Tepp, Marite Bradshaw, Alexander P. Gardner, Rebecca L. Kaufman, Joseph T. Barbieri, Sabine Pellett

**Affiliations:** 1 Department of Bacteriology, University of Wisconsin, Madison, WI 53706, USA; 2 Department of Microbiology and Molecular Genetics, Medical College of Wisconsin, Milwaukee, WI 53226, USAjtb01@mcw.edu (J.T.B.)

**Keywords:** botulinum neurotoxin, clostridium botulinum, SV2C, receptor, potency, BoNT/A4, BoNT, subtypes

## Abstract

Botulinum neurotoxin subtype A4 (BoNT/A4) is ~1000-fold less potent than BoNT/A1. This study addresses the basis for low BoNT/A4 potency. Utilizing BoNT/A1-A4 and BoNT/A4-A1 Light Chain-Heavy Chain (LC-HC) chimeras, HC-A4 was responsible for low BoNT/A4 potency. Earlier studies showed BoNT/A1-receptor binding domain (Hcc) bound a β-strand peptide (556–564) and glycan-N^559^ within Luminal Domain 4 (LD4) of SV2C, the BoNT/A protein receptor. Relative to BoNT/A1, the Hcc of BoNT/A4 possesses two amino acid variants (D^1141^ and N^1142^) within the β-peptide binding interface and one amino acid variant (R^1292^) located near the SV2C glycan-N^559^. Introduction of BoNT/A4 β-strand peptide variant (D^1141^ and N^1142^) into BoNT/A1 reduced toxin potency 30-fold, and additional introduction of the BoNT/A4 glycan-N^559^ variant (D^1141^, N^1142^, and R^1292^) further reduced toxin potency to approach BoNT/A4. While introduction of BoNT/A1 glycan-N^559^ variant (G^1292^) into BoNT/A4 did not alter toxin potency, additional introduction of BoNT/A1 β-strand peptide variants (G^1141^, S^1142^, and G^1292^) resulted in potency approaching BoNT/A1 potency. Thus, outcomes from these functional and modeling studies indicate that in rodent models, disruption of Hcc -SV2C β-peptide and -glycan-N^559^ interactions mediate low BoNT/A4 potency, while in human motor neurons, disruption of Hcc-SV2C β-peptide alone mediates low BoNT/A4 potency, which link to a species-specific variation at SV2C^563^.

## 1. Introduction

Botulinum neurotoxins (BoNTs), the causative agents of human and animal botulism, constitute a large family of protein toxins produced by *Clostridium botulinum* and some strains of related species [1]. The BoNT family includes seven immunologically distinct serotypes (A-G) that are neurotoxic in humans and vertebrate animals [2]. Within these serotypes, over 40 distinct subtypes have been described, with current nomenclature guidelines defining a subtype as having at least 2.6% amino acid variance to other subtypes within the same serotype; however, additional subtype variants exist that exhibit fewer than 2.6% amino acid variation [3]. Only few of the BoNT subtypes have been functionally characterized and the consequences of most naturally occurring amino acid variations on BoNT toxicity to humans and vertebrates is unknown.

BoNTs are AB-type bacterial toxins, consisting of a 50 kDa light chain (LC) domain, which is a zinc-dependent endopeptidase, and a 100 kDa heavy chain (HC) that aids in specific neuronal cell entry [4,5]. The HC can be subdivided into three structural and functional domains, the translocations domain (H_N_), the N-terminal portion of the receptor binding domain (H_CN_), and the C-terminal portion of the receptor binding domain, which contains the ganglioside- and protein-receptor binding domains (H_CC_). In *C. botulinum*, BoNTs are produced as a single chain 150 kDa protein, which is post-translationally converted into a more active di-chain form by proteolytic cleavage between the LC and HC, which are linked by a disulfide bond [4].

One of the hallmarks of BoNTs is their extraordinary potency in motor neurons. A lethal dose of BoNT/A1 is only 1–2 ng/kg for humans by intravenous administration [6]. This high neuron-specific potency makes BoNTs significant toxins and potential bioweapons, but also valuable biopharmaceuticals, enabling local injection of extremely low doses of the toxin to achieve a localized therapeutic effect [5,7,8,9,10,11,12,13]. Currently, only two BoNT subtypes are used pharmaceutically, BoNT/A1 and to a lesser extent BoNT/B1.

Past research has revealed that subtypes of one serotype can have distinct characteristics. BoNT/A subtypes differ in neuronal cell entry, enzymatic activity of the LC, intracellular trafficking, and potency [14,15,16,17,18,19,20,21,22,23,24,25,26,27,28,29]. The gene encoding BoNT/A4 is localized on a plasmid that also carries the gene encoding BoNT/B in the bivalent toxin producing *Clostridium botulinum* strain 657, showing the potential diverse genetic organization of these potent pathogens [30]. Isolated BoNT/A4 has an ~1000-fold lower potency in mice and human neurons [17,18]. Endopeptidase studies indicate BoNT/A4 LC has slightly reduced catalytic activity compared to BoNT/A1 [18,31], with holotoxin assays indicating a five- to >forty-fold reduction in SNAPtide catalysis depending on assay conditions [18], and recombinant LC studies indicating a 100-fold reduced cleavage efficiency (k_cat_/K_m_) [31]. However, *E. coli* produced BoNT/A4 LC was not fully soluble, and introduction of a mutation (I^264^R) to increase solubility also increased enzymatic efficiency to levels within two-fold of LC/A1 [31]. Introduction of the same mutation into the holotoxin did not affect in vivo potency of BoNT/A4 [17], indicating the lower enzymatic activity observed in endopeptidase assays may be due to insolubility of recombinant BoNT/A4 LC domain rather than a biologic property of the holotoxin. Thus, the ~1000-fold lower potency of BoNT/A4 cannot be explained by differences in catalytic activity of the LC.

A systematic mutational study of BoNT/A1 addressed properties of the protein receptor-binding domain. Using a series of BoNT/A1 holotoxin mutants produced in *E. coli*, an earlier study revealed that the single amino acid alteration G^1292^R, which naturally occurs in BoNT/A4, negatively affected protein receptor binding and reduced toxicity 300-fold as measured by the mouse hemidiaphragm assay [32], indicating the receptor binding domain of BoNT/A4 may be involved in lower potency. While intriguing, these analyses did not elucidate the molecular basis underlying the ~1000-fold reduction in BoNT/A4 potency compared to BoNT/A1.

In this study, the basis for the low potency of BoNT/A4 was determined. Initial experiments, using BoNTA1-A4 chimeras, showed A4-HC was responsible for low BoNT/A4 potency. Structural alignment studies identified two BoNT/A4 amino acid variants D^1141^, N^1142^ potentially involved in binding to the β-peptide of the Luminal Domain 4 (LD4) of SV2C and R^1292^ potentially interacting with the glycan-N^599^ within the β-peptide. Using a mouse bioassay and cell-based assays, the three amino acid variations D^1141^, N^1142^, and R^1292^ of BoNT/A4 were shown to be responsible for the lower potency of this subtype.

## 2. Results

### 2.1. Low Potency of BoNT/A4 Is Observed in a Mouse Bioassay, Rat Spinal Cord Cells, and Human Motor Neurons

Initial experiments assessed the relative sensitivity of several model systems to BoNT/A1 and BoNT/A4 prototypes. In a six-day mouse bioassay, BoNT/A1 was ~1300-fold more potent than BoNT/A4 (Table 1). Measuring SNAP-25 cleavage as an outcome for BoNT/A sensitivity (Table 1), human motor neurons showed an ~8000-fold differential sensitivity between BoNT/A1 and BoNT/A4, while rat spinal cord cells showed an ~2000-fold differential sensitivity between BoNT/A1 and BoNT/A4. This assay-specific difference may be due to the more sensitive human motor neurons cells, which were ~14-fold more sensitive to BoNT/A1 than rat spinal cord cells as also previously observed [33,34,35,36].

### 2.2. Low Potency of BoNT/A4 Is due to the A4-HC Domain

**Figure 1:**
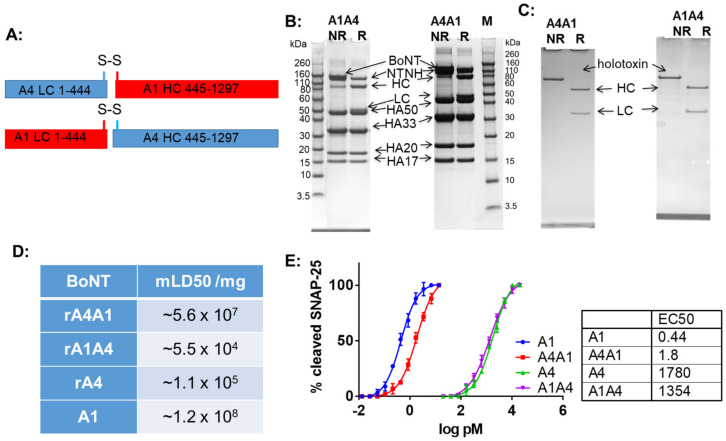
Purified BoNT/A1 and /A4 LC-HC chimeras and biologic activity. The chimeric BoNTs were expressed in *C. botulinum* strain Hall A hyper/tox^-^ and purified using biochemical methods. (**A**) schematic showing the chimera design. (**B**) Comassie stained SDS-PAGE gels of nonreduced (NR) and reduced (R) samples of isolated BoNT/A1A4 and /A4A1 toxin complexes. The complexing proteins are indicated. (**C**) Comassie stained SDS-PAGE gels of nonreduced (NR) and reduced (R) samples of purified BoNT/A4A1 and BoNT/A1A4. (**D**) Specific toxicity of wt and chimeric BoNT/A1 and /A4 in mice. (**E**) Potency of wt and chimeric BoNT/A1 and /A4 in cultured human induced pluripotent stem cell derived neurons. *n* = 3.

To determine whether the lower potency of BoNT/A4 relative to BoNT/A1 was due to a defect in the enzymatic LC domain or in the cell entry HC domain, recombinant tag-less, chimeric BoNTA1-A4 and BoNTA4-A1 (LC-HC) holotoxins were constructed (Figure 1A). As observed for recombinant BoNT/A4 expressed in the same *C. botulinum* strain Hall A hyper/tox^-^ [17], the recombinant chimeras BoNTA1-A4 and BoNTA4-A1 were eluted in a single peak from DEAE-Sephadex A-50 column at pH 5.5 together with the nontoxic complexing proteins, indicating that the chimeric proteins formed complexes with the BoNT/A1 complexing proteins produced by the expression strain (Figure 1B). BoNTA1-A4 and BoNTA4-A1 complexes were purified to 150-kDa BoNTA1-A4 and BoNTA4-A1. SDS-PAGE analysis showed high purity and processing of chimeric BoNTA1-A4 and BoNTA4-A1 to the di-chain as indicated by the reduction of the 150-KDa proteins to 100-kDa and 50-kDa proteins upon DTT addition (Figure 1C). This indicated that the assembly and activation of BoNTA1-A4 and BoNTA4-A1 proceeded as normal for BoNTs produced in clostridia. Examination of the specific activity in mice showed that BoNTA4-A1 had similar potency (within ~2-fold) as BoNT/A1 [18] and BoNTA1-A4 had similar potency (within ~2-fold) to BoNT/A4 [17] (Figure 1D). Potency determinations in human iPSC-derived neurons confirmed similar activities for BoNT/A1 and BoNT/A4-A1 (within ~4 fold), and for BoNT/A4 and BoNT/A1-A4 (equivalent) (Figure 1E). Together, these results demonstrated that a rate-limiting step of BoNT/A4 toxicity localizes to the A4-HC.

**Figure 2:**
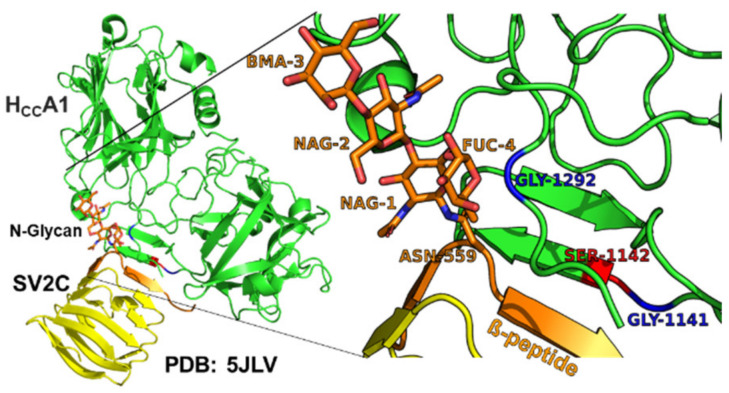
Three BoNT/A1-BoNT/A4 amino acid variants (G^1141^D, S^1142^N, and G^1292^R) located at the interface of BoNT/AH_CC_ and β-peptide (brown) within LD4 of SV2C (PDB: 5JLV). The G^1141^D, S^1142^N, and G^1292^R variants of BoNT/A1 and BoNT/A4, respectively, were chosen for assessment, since Hcc/A1 G^1141^ and S^1142^ were adjacent to the β-peptide of Luminal Domain 4 (LD4) of SV2C, and Hcc/A1 G^1292^ was located adjacent to the glycan network of glycosylated-N^559^ (brown) within the β-peptide of LD4 of SV2C. Modeling of Hcc/A4 showed the location of D^1141^ (red), N^1142^ (blue), and G^1292^ (blue) PDB: 6F0P.

Sequence alignments showed three amino acid variants in BoNT/A4 within the SV2C Luminal Domain 4 at the H_CC_-SV2C binding interface. While the secondary and tertiary BoNT/A subtype structures are conserved [37,38], primary amino acid identity between BoNT/A1 and BoNT/A4 is ~89%, with 90% identity in the LC, ~85% in the HC translocation domain (H_CN_), and ~92% in the HC receptor binding domain (H_CC_). BLASTP identified 36 amino acid H_CC_ variants between BoNT/A1 and BoNT/A4, with eight of those unique to BoNT/A4 compared to all other known BoNT/A subtypes (Supplementary Figure S1). Structural modeling based on solved crystal structures indicated two of the unique H_CC_ amino acid variants (G^1141^D and S^1142^N) of BoNT/A4 were adjacent to the H_CC_ binding interface with the β-peptide of Luminal Domain 4 (LD4) of SV2. Residue G^1292^R was positioned behind the SV2C binding interface and adjacent to the glycan network of glycosylated-N559 within the β-peptide of LD4 of SV2C (Figure 2) and has previously been described as essential for SV2C binding via N559-glycan interaction [32,39]. For clarity, ^1141^, and ^1142^ will be described as the β-peptide variants, while ^1292^ is described as the glycan N^559^ variant.

### 2.3. The β-Peptide Variants and Glycan N^559^ Variant of BoNT/A4 Together Modulate Toxin Potency

**Figure 3:**
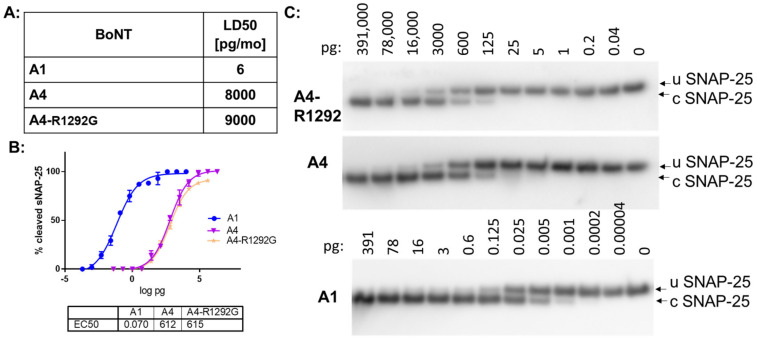
Mutational analysis of BoNT/A1 and BoNT/A4 protein receptor binding. (**A**) BoNT/A1, BoNT/A4, and BoNT/A4-R^1292^G variant were tested in the mouse bioassay. Four mice each were injected with an IP dose of the indicated BoNT/A and observed for 6 days, establishing a 50% potency, expressed as LD50 (pg/mouse). (**B**) Biologic activity of BoNT/A4-R^1292^G (A1-1M). Human iPSC derived motor neurons were exposed to the toxins for 48 h, and cell lysates analyzed for SNAP-25 cleavage by Western blot. The percent cleaved SNAP-25 was determined by densitometry, and average values and standard deviations were plotted in Prism6, *n* = 3. EC50 values were derived from a nonlinear four-parameter regression curve fit in Prism6. (**C**) Representative Western blots of the cell-based assay. U SNAP-25 (uncleaved SNAP-25), c SNAP-25 (cleaved SNAP-25).

Introduction of BoNT/A1 glycan-N^559^ variant (G^1292^) into BoNT/A4 did not alter toxin potency in mice and in cultured human motor neurons (Figure 3). This indicates that the R^1292^ in BoNT/A4 by itself does not determine lower potency of BoNT/A4. Introduction of both the BoNT/A1 β-strand peptide variants (G^1141^ and S^1142^) and the glycan-N^559^ variant (G^1292^) into BoNT/A4 resulted in a mouse LD50 of 15pg/mouse, approaching the potency of BoNT/A1 (Table 2).

**Figure 4:**
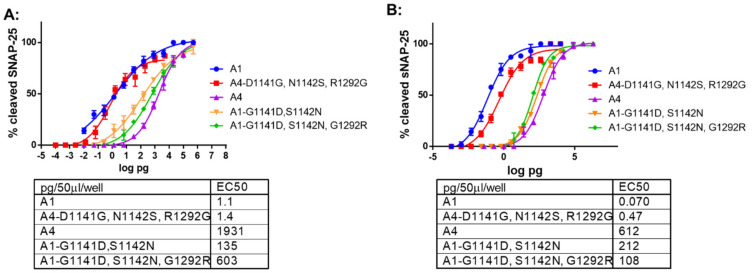
Neuronal cell-based assays of BoNT/A1 and /A4 variant proteins. (**A**) Potency of BoNT/A1 and BoNT/A4 variants in primary rat spinal cord neurons. (**B**) Potency of BoNT/A1 and BoNT/A4 variants in human iPSC derived motor neurons. Neurons were exposed to the toxins for 48 h, and cell lysates analyzed for SNAP-25 cleavage by Western blot. The percent cleaved SNAP-25 was determined by densitometry, and average values and standard deviations were plotted in GraphPad Prism 6, *n* = 3. EC50 values were derived from a nonlinear four-parameter regression curve fit in GraphPad Prism 6 (Dotmatics, San Diego, CA, USA).

Similarly, in primary rat spinal cord cells and human iPSC derived motor neurons assays, toxin potency of BoNT/A4 (G^1141^, S^1142^, and G^1292^) was ~1400 fold greater than BoNT/A4 and approached that of BoNT/A1 (Figure 4, Table 2). While the primary rat spinal cord cells assay revealed no difference in potency between BoNT/A1 and BoNT/A4 (G^1141^, S^1142^, and G^1292^), BoNT/A1 remained ~6-fold more potent in the human iPSC derived motor neurons. These findings show that amino acid variants of BoNT/A4 adjacent to the LD4 β-strand peptide of SV2C (D^1141^ and N^1142^) and glycan-N^559^ (R^1292^) are the primary defects responsible for the low BoNT/A4 potency.

### 2.4. The β-Peptide and Glycan N^559^ Variants Modulate the Potency of BoNT/A1

In a mouse bioassay, introduction of BoNT/A4 β-strand peptide residues (D^1141^ and N^1142^) into BoNT/A1 reduced toxin potency ~30 fold, while introduction of both the BoNT/A4 β-peptide variants (D^1141^ and N^1142^) and the glycan-N559 variant (R^1292^) into BoNT/A1 reduced toxin potency ~200-fold, approaching but not reaching the lower potency of BoNT/A4 (Table 2). In the primary rat spinal cord cells assay, a corresponding sequential decrease in toxin potency was observed for the same mutants, with a ~100-fold decreased potency for BoNT/A1 (D^1141^ and N^1142^) and a ~600-fold decreased potency for BoNT/A1 (D^1141^, N^1142^, and R^1292^). These findings show that amino acid variants of BoNT/A4 adjacent to the LD4 β-strand peptide of SV2C (D^1141^ and N^1142^) and glycan-N^559^ (R^1292^) are the primary defects responsible for the low BoNT/A4 potency. Interestingly, using human motor neurons in a cell-based assay, both the introduction of BoNT/A4 β-strand peptide variants (D^1141^ and N^1142^) into BoNT/A1 and introduction of the BoNT/A4 β-peptide and glycan-N559 variants (D^1141^, N^1142^, and R^1292^) into BoNT/A1 reduced toxin potency ~1500–2000-fold and within 4–6-fold of BoNT/A4 potency (Figure 4). Thus, unlike our observations of the mouse bioassay and rat spinal cord cells, addition of both the β-peptide variants (D^1141^ and N^1142^) and the glycan-N^559^ variant (R^1292^) into BoNT/A1 did not decrease potency beyond the effect of introducing the β-peptide variants (D^1141^ and N^1142^) into BoNT/A1. The limited effect of the addition of R^1292^, in addition to the β-peptide variants (D^1141^ and N^1142^), may reflect a more dominant effect of the β-peptide variants (D^1141^ and N^1142^) at the H_CC_:Sv2 interface in the human motor neuron cell model than observed either in the mouse bioassay or rat spinal cord cells. Overall, introducing the β-peptide variant residues D^1141^ and N^1142^, and the glycan-N^559^ variant residue R^1292^, modifies BoNT/A1 to elicit a reduced BoNT/A4-like potency, and introducing the complementary A1 residues (G^1141^, S^1142^, and G^1292^) modifies BoNT/A4 to elicit a BoNT/A1-like potency. This supports roles for (D^1141^ and N^1142^) within the β-peptide and R^1292^ involved in glycan-N^559^ interactions as being the primary amino acids that establish the low potency of BoNT/A4.

## 3. Discussion

Using a mouse bioassay, primary rat spinal cord cells, a human motor neurons cell model, and computational approaches, this study resolved the functional basis for low BoNT/A4 potency. BoNT/A1-A4 and BoNT/A4-A1 (Light Chain-Heavy Chain, LC-HC) chimeras in the mouse bioassay showed HC-A4 to be responsible for low BoNT/A4 potency (Figure 1). This is in agreement with previously published data on catalytic activity of reduced BoNT/A4 holotoxin and soluble rLC/A4, which indicated only about 2–5-fold reduced catalytic activity compared to BoNT/A1 or rLC/A1, respectively [18,31].

Structural modeling and mutational analyses targeted the structural and functional HC domain that determines BoNT/A4 low potency. As confirmed by recent crystallography studies, the translocation domain (H_N_) and ganglioside binding pockets of BoNT/A1 and BoNT/A4 are identical [37]. Our efforts focused on the SV2 binding pocket within the receptor binding domain. In fact, structural analysis indicated that of the five amino acid residues unique to BoNT/A4 in the C terminus of H_CC_ (residues ^1091−1296^), three BoNT/A4 amino acid variants were located within the predicted SV2 binding interface (Figure 2A and  Figure S1). Earlier studies showed BoNT/A-receptor binding domain (H_CC_) interacts with a β-strand peptide (^556−564^) and the glycan of N^559^ within Luminal Domain 4 (LD4) of SV2C, the BoNT/A protein receptor, whereby there is significant plasticity for receptor recognition [32,39,40,41,42,43,44]. Relative to BoNT/A1, BoNT/A4 possessed two amino acid variants (D^1141^ and N^1142^) adjacent to the β-peptide and one amino acid variant (R^1292^) located near glycan-N^559^ [37] (Supplementary Figure S1.)

**Figure 5:**
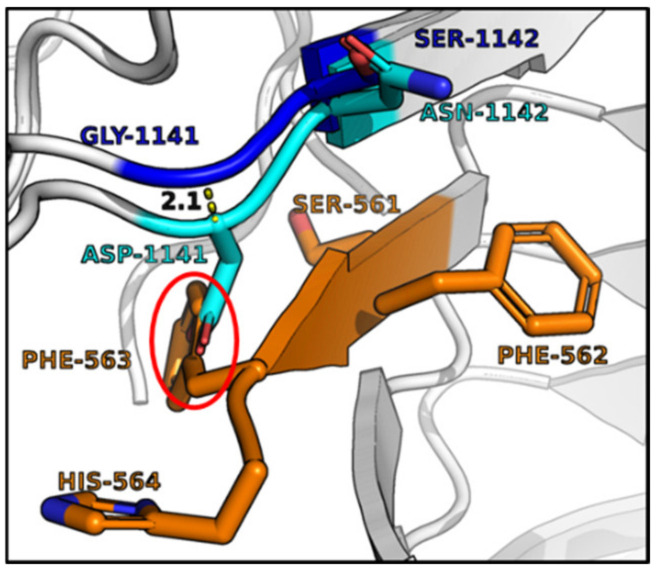
The anion of D1141 disrupts organization of the pi network of Phe563 in a modeled HCC/A4-LD4 of SV2C complex. The corresponding region of HCC/A4 (PDB:6F0P) was overlapped on the cocrystal of HCC/A1-LD4 of SV2C (PDB:5JLV). Note the extension of the carboxylate of D1141 into the pi network of LD4-Phe563 (red circle), while A4-Asn1142 and A1-Ser1142 are distanced from the SV2C β-strand.

Our functional analyses of site-specific mutated BoNT/A4, exchanging the three amino acid residues unique between BoNT/A1 and BoNT/A4 (D^1141^G, N^1142^S, and R^1292^G), respectively, confirmed their primary role in eliciting the ~1000-fold reduced toxin potency of BoNT/A4 (Table 2, Figure 4). In the mouse bioassay and rat spinal cord cells, introduction of BoNT/A4 β-strand peptide variant (D^1141^ and N^1142^) into BoNT/A1 reduced toxin potency ~30-fold and ~100-fold, respectively. Introduction of BoNT/A4 β-strand peptide and glycan-N^559^ variants (D^1141^, N^1142^, and R^1292^) into BoNT/A1 reduced toxin potency ~200 fold in mice to approach BoNT/A4, while introduction of BoNT/A1 β-strand peptide and glycan-N^559^ variants (G^1141^, S^1142^, and G^1292^) into BoNT/A4 resulted in potency approaching BoNT/A1 potency (Table 2, Figure 4). Thus, in the mouse bioassay and rat spinal cord cells, BoNT/A4-H_CC_ amino acid variants juxtaposed with the SV2C β-peptide and glycan-N^559^ contributed to low BoNT/A4 potency. In contrast, in human motor neurons, BoNT/A1(D^1141^ and N^1142^) alone decreased BoNT/A1 potency approaching that of BoNT/A4, while the addition of the R^1292^ mutation did not further decrease potency as was observed in the mouse bioassay and rat spinal cord cells. This observation may reflect a greater effect of D^1141^ and N^1142^ alone at the β-peptide interface of LD4 in human neurons specifically. There are two amino acid alterations in the SV2C LD4 β-peptide that BoNT/A binds, including F^563^ that is unique to human SV2C, where rat and mouse SV2C as well as human SV2A and SV2B encode L^563^ (Supplementary Figure S2). Modeling BoNT/A4 H_CC_ with the SV2C β-peptide showed that D^1141^ of H_CC_/A4 inserts into the phenyl ring R-group of SV2C F^563^ (Figure 5), where the close proximity of the negatively charged aspartic acid and the aromatic phenylalanine may disrupt binding via anion–pi pair repulsion [45], leading to a steric distortion of the Hcc-LD4 binding interface. These findings provide a basis for a SV2C-LD4 R^563^A mutation that reduced the capacity of A1-Hcc to bind SV2C [41]. This raises the question of whether positioning and/or length of the BoNT/A H_CC_ and the β-peptide interactions differ in humans versus rodents, due to alterations in the SV2C LD4 β-peptide that BoNT/A binds and/or the R-group compositions of BoNT/A subtypes at the β-peptide LD4 interface (Supplementary Figure S2).

**Figure 6:**
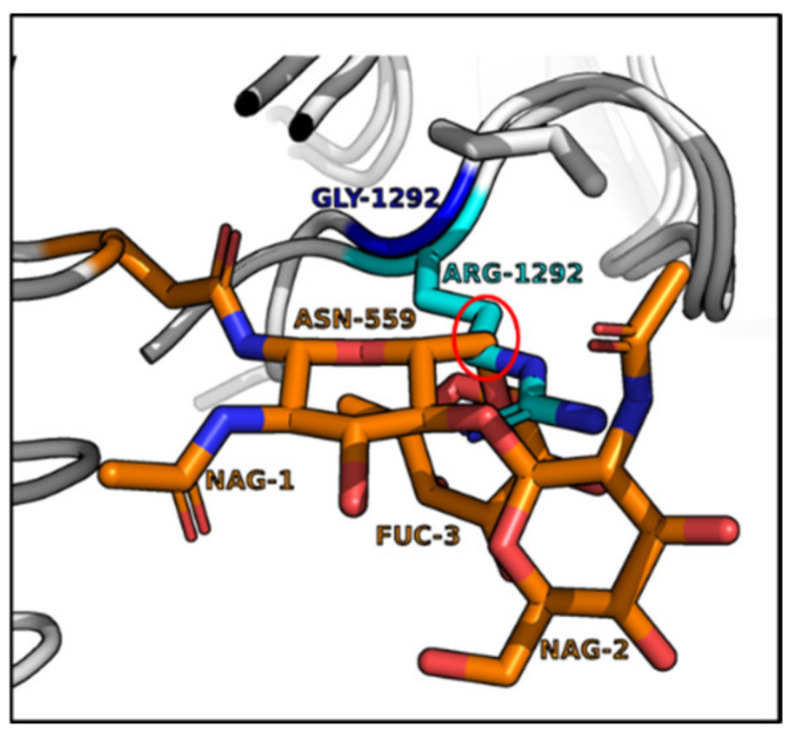
The guanidinium ring of R1292 disrupts the glycan-N559 network in a modeled HCC/A1(G1292R)-LD4 of SV2C complex. The corresponding region of HCC/A4 (PDB:6F0P) was overlapped on the cocrystal of HCC/A1-LD4 of SV2C (PDB:5JLV). Note the extension of the guanidinium ring of R1292 into the glycanN559 network (red circle).

These data agree with the well-documented direct interaction of Hcc/A with the SV2C LD4 β-strand [32,39,40,41,42,43,44], and suggest a central role for the G^1292^ of BoNT/A1 for biologic function. This is consistent with previous data demonstrating a strong (300-fold) decrease in toxicity of *E. coli* produced BoNT/A1(G^1292^), as measured by the rat hemidiaphragm assay [32]. The data are also consistent with analyses of crystal structures of BoNT/A1, /A2, and /A4 H_CC_ alone or in complex with the Luminal Domain 4 of SV2C indicating a potential role for residue^1292^ in interaction with glycan-N^559^ [38,39,40,41]. In these studies, the G^1292^-glycan-N^559^ interaction was essential to strengthen the H_CC_-β-peptide interaction for optimal binding of BoNT/A1 to SV2C [32], leading to the conclusion that the substitution of this residue with arginine in BoNT/A4 weakens this interaction and thus weakens SV2C binding. While the G^1292^ of BoNT/A1 spatially aligns well adjacent to the glycan, the R^1292^ of BoNT/A4 extends into the glycan structure (Figure 6). In rodent models, BoNT/A4 R^1292^ may thereby spatially interfere with the alignment of the H_CC_ with the SV2C β-peptide, potentially leading to distortion of the binding interface. This interference does not appear in the human model tested, where introduction of the BoNT/A1 glycan-N^559^ variant (G^1292^) into BoNT/A4 by itself did not alter toxin potency (Figure 3). This may indicate the instability engendered by R^1292^G may not be relevant if the H_CC_ has not engaged with the β-peptide of LD4 in human SV2C, where interactions of BoNT/A with the β-peptide of LD4 and the glycan-N^559^ may be functionally ordered events. Thus, the G^1292^R disruptive phenotype may be a property specific to rodent models. The observed phenotype of BoNT/A1(D^1141^, N^1142^, and R^1292^) and BoNT/A1(D^1141^ and N^1142^) but not BoNT/A4(G^1292^) supports the sequential binding of BoNT/A to the LD4 β-peptide followed by an interaction with the LD4 glycan-N^559^(Figure 4 and Table 2).

Our study utilized three biologic models to analyze the effects of the BoNT/A1 and /A4 amino acid exchanges on potency in mice, primary rat spinal cord cells, and human iPSC derived motor neurons. Effects were observed to be greatest in the human motor neurons followed by rat spinal cord cells, then mice. This in part reflects the greater sensitivity of the cell models, but we also observed unique sensitivity of the human iPSC derived neurons model. Specifically, the lack of an additive effect of the R^1292^ when added to the BoNT/A1(D^1141^ and N^1142^) in human motor neuron versus the additive effect observed in rat spinal cord cells and mice (Table 2, Figure 4) may reflect a greater effect of D^1141^ and N^1142^ alone at the β-peptide interface of LD4 in human neurons. Future biophysical and structural studies directly examining species and isoform specific SV2 binding with H_CC_s containing the BoNT/A4 residue alterations are warranted to further examine mechanisms determining BoNT/A potency.

In summary, the data presented here demonstrate that three naturally occurring amino acid variations primarily determine the ~1000-fold reduction in BoNT/A4 potency as compared to BoNT/A1. The three amino acid residues localize to the SV2 binding interface, with predicted direct interaction with SV2 for D^1141^/N^1142^, and either indirect structural distortion or direct interaction with the glycan-N^559^ of glycosylated SV2 with R^1292^. Thus, BoNT/A4 has lower potency due to the altered SV2 protein receptor, BoNT/A4 interaction, adding to our understanding of the previously observed plasticity in BoNT/A-SV2C interactions [40,46]. Understanding the molecular mechanisms underlying BoNT potency will enable future developments of novel BoNT based pharmaceuticals as well as countermeasures.

## 4. Materials and Methods

### 4.1. Biosafety, Biosecurity, and Ethics

Pellett laboratory and personnel are registered with the CDC Select Agent Program for research involving botulinum neurotoxins and botulinum neurotoxin-producing strains of clostridia. The research program, procedures, occupational health plan, documentation, security, and facilities are closely monitored by the University of Wisconsin—Madison Biosecurity Task Force, University of Wisconsin—Madison Office of Biological Safety, the University of Wisconsin Select Agent Program, and at regular intervals by the CDC and the Animal and Plant Health Inspection Service (APHIS) as part of the University of Wisconsin—Madison Select Agent Program. All personnel have undergone suitability assessments and completed rigorous and continuing biosafety training, including biosafety level 3 (BSL3) and select agent practices before participating in laboratory studies involving botulinum neurotoxins and neurotoxigenic *C. botulinum* strains. All recombinant DNA protocols for the construction of the recombinant BoNT genes and their expression in *C. botulinum* strains have been approved by the University of Wisconsin Institutional Biosafety Committee (IBC) with specific experiments approved by the Division of Select Agents and Toxins at the CDC (protocol # B00000934, approved 7 January 2021). A dual use research of concern (DURC) risk mitigation plan has been established and approved by the University of Wisconsin—Madison Select Agent Program and NIAID for these experiments. Preparation of the recombinant BoNT gene constructs was performed under biosafety level 2, while experiments involving transfer of gene expression vectors into the *C. botulinum* expression host strain and purification of the recombinant BoNT were performed in a biosafety level 3 (BSL3) facility, as described in the CDC/NIH documents and in accordance with Select Agent regulations.

Animal studies involving BoNT select agents were approved by the University of Wisconsin–Madison Institutional Animal Care and Use Committee (IACUC, protocol number M006326-A02, approval date 27 April 2020).

### 4.2. Reagents

Oligonucleotide primers were synthesized by IDT (Coralville, IA) and are listed in Table 3. PCR reactions were performed using Phusion High-Fidelity Master mix with HF buffer or Phusion Hot Start Flex 2X Master mix (New England Biolabs, Ipswich, MA, USA). Restriction endonucleases, Quick CIP (calf alkaline phosphatase), DNA ligase, and competent *E. coli* DH10ß cells were purchased from New England Biolabs (Ipswich, MA, USA). Mutations were introduced into the BoNT genes using QuikChange Lightning Multi site-directed mutagenesis kit (Agilent Technologies, Santa Clara, CA, USA). The antibiotics carbenicillin, chloramphenicol, kanamycin, thiamphenicol, erythromycin, and cycloserine were purchased from Sigma-Aldrich (St. Louis, MO). Media reagents were purchased from Sigma-Aldrich (St. Louis, MO, USA) or DIFCO-BD biosciences (Franklin Lakes, NJ, USA).

### 4.3. Bacterial Strains and Media

*E. coli* strains were grown at 37 °C in LB media or on LB agar plates supplemented with the appropriate antibiotics for plasmid selection. *E. coli* strain CA434 (kindly provided by N. Minton, University of Nottingham, UK) served as the donor strain for conjugal expression vector transfer from *E. coli* to *C. botulinum*. Clostridia were grown in 5% Trypticase peptone, 0.5% Bacto peptone, 0.4% glucose, 2% yeast extract, and 0.1% L-cysteine HCl [pH 7.3–7.4]) (TPGY) liquid media or TPGY agar plates. For toxin production, clostridia were grown in Toxin Production Medium (TPM; 2% N-Z-Case™ TT, 0.5% glucose, 1% yeast extract [pH 7.3]). Clostridia were grown statically in nitrogen-flushed Hungate tubes or glass bottles at 35 °C, and cell manipulations were conducted in an anaerobic chamber with an atmosphere of 80% N_2_, 10% CO_2_, and 10% H_2_ (Forma Anaerobic System, Marietta, OH, USA). Antibiotics were used in the following concentrations: in *E. coli*, carbenicillin at 50 µg/mL, kanamycin at 50 µg/mL, and chloramphenicol at 25 µg/mL in agar plates and 12.5 µg/mL in liquid media; in *C. botulinum*, cycloserine at 250 µg/mL, thiamphenicol at 15 µg/mL, and erythromycin at 20 µg/mL.

### 4.4. Botulinum Neurotoxins

The 150-kDa BoNT/A1 was isolated from the wild type *C. botulinum* strain Hall A hyper as previously described [47], while recombinant 150-kDa BoNT/A4 was expressed in the atoxic *C. botulinum* strain Hall A-hyper/tox^-^ and isolated as previously described [17].

To design hybrid BoNT/LCA1-HCA4 and BoNT/LCA4-HCA1, individual light chain (LC, aa 1–444) and heavy chain (HC, aa 445–1297) gene regions encoding botulinum toxin serotype subtype A1 (*bont*/A1) (GenBank accession number AF461540) and *bont*/A4 (GenBank accession number CLJ_0004) were amplified by PCR using total genomic DNA isolated from *C. botulinum* strains Hall A-hyper and 657Ba as templates, respectively. PCR primers (Table 3) were designed to amplify the LC and HC gene fragments from each subtype and contained additional sequences from the other subtype at the LC-3′ and 5′-HC ends to enable generation of a seamless junction between the two different subtype gene regions using splicing by overlap extension (SOEing) PCR. The internal NdeI site in the HC/A4 gene fragment was eliminated by a QuikChange reaction using primer A4-Nde2029 to introduce a silent mutation. Nucleotide sequence of BoNT/LCA1-HCA4 and BoNT/LCA4-HCA1 was verified by Sanger DNA sequencing (UW–Madison, Biotech Center).

To create BoNT/A1 and BoNT/A4 with specific amino acid mutations, the entire BoNT/A1 gene was amplified by PCR using total genomic DNA isolated from *C*. *botulinum* strain Hall A-hyper (GenBank accession number AF461540) and Fusion Hot Start Flex 2x Master mix according to the manufacturer’s instructions (New England Biolabs). Generation of recombinant *bont*/A4 has been described previously [17]. Restriction enzyme sites for *Nde*I and *Nhe*I were included into 5′ and 3′ PCR primers, respectively (Table 3). Specific amino acid mutations (for BoNT/A1: G^1141^D, S^1142^N, and G^1292^R, and for BoNT/A4: D^1141^G, N^1142^S, and R^1292^G) were introduced into the rBoNT/A1 or rBoNT/A4 genes, using the QuikChange Lightning Multi site-directed mutagenesis kit according to the manufacturer’s instructions (Agilent Technologies) and primers shown in Table 3. Introduction of nucleotide substitutions was confirmed by sequencing the mutated genes (UW–Madison, Biotech Center, Madison, WI, USA).

All recombinant mutant and hybrid BoNT genes were inserted into clostridial expression vector pMTL83152 [48]. The expression constructs were transferred into the nontoxigenic *C. botulinum* expression host strain Hall A hyper/tox- by conjugation from an *E. coli* CA434 donor strain as previously described [17]. The presence of the expression plasmid in *C. botulinum* Hall A-hyper/tox^−^ was confirmed by plasmid isolation from the conjugated strain followed by restriction digest of the plasmid and sequencing of the mutated or hybrid genes. The hybrid or mutant BoNTs were produced in the strain Hall A-hyper/tox- grown in TPM supplemented with 15 µg/mL thiamphenicol for 4 days at 37 °C, followed by purification of the 150-kDa protein toxins as previously described [47]. Purified toxins were stored in phosphate buffered saline, 40% glycerol at −20 °C. Toxin concentrations were determined by absorbance measurement at A^278^ and an extinction coefficient of 1.63 for 1 mg/mL in a 1 cm light path [49].

### 4.5. Neuronal Cell Based Assay

Human hiPSC-derived motor neurons were purchased from Fujifilm Cellular Dynamics (Madison, WI, USA). The cells were seeded and fed as recommended by the manufacturer using the media supplied with the cells and cultured for at least 14 days (14 DIV) prior to the BoNT assays. Primary rat spinal cord cells were prepared from E15 Sprague Dawley rat pups (Envigo, Madison, WI, USA) and maintained as previously described [50,51,52]. Cells were maintained at least 14 DIV before use in the BoNT assay.

For the cell-based assay, cells were exposed to the indicated concentrations of BoNTs in 50 μL of each respective neuronal medium. All toxins compared to each other were tested in parallel using the same cell batches. After a 48 h exposure time, the toxin solution was removed, and cells were lysed in 50 μL of 1× lithium dodecyl sulfate sample buffer (Life Technologies, Carlsbad, CA, USA). Cell lysates were analyzed by Western blot for SNAP-25 cleavage as previously described [51,52]. Western blots were imaged on an Azure c600 imaging system and cleaved and uncleaved SNAP-25 bands were quantified by densitometry using Azure spot software version 2.0.062 (Azure Biosystems, Dublin, CA, USA). Data plots and EC50 values or estimates were generated in GraphPad prism 6 software (San Diego, CA, USA) using a nonlinear four parameter ordinary fit curve fit. All samples were tested in triplicate and a negative control without toxin was included.

### 4.6. Specific Toxin Activity Determination

BoNT specific activity was determined by a mouse bioassay (MBA) [53,54]. Serial dilutions of the respective toxins were prepared in 30 mM sodium phosphate buffer pH 6.3 containing 0.2% gelatin (GelPhos buffer) and administered by intraperitoneal injection into six groups of four female mice (0.5 mL/mouse). Mice were observed for up to 6 days. Specific activity was determined based on the method of Reed and Muench [55] and expressed as pg toxin/LD_50_.

## Figures and Tables

**Table 1:** Potency of BoNT/A1 and BoNT/A4 in mice and human motor neurons and rat spinal cord cells.

BoNT	Mouse LD50 [pg/Mouse]	EC50 MN [pg/Well]	EC50 RSC [pg/Well]
A1 (wt)	6	0.07	1
A4 (wt)	8000	612	1943

MN: hiPSC derived motor neurons, RSC: primary rat spinal cord cells.

**Table 2:** Potency of BoNT mutants in mice and neuronal cell cultures.

BoNT	Mouse LD50 [pg/Mouse]	EC50 MN [pg/Well]	EC50 RSC [pg/Well]
A1 wt	6	0.07	1.1
A4 wt	8000	612	1931
A4-D1141G, N1142S, R1292G	15	0.47	1.4
A1-G1141D, S1142N	175	212	135
A1-G1141D, S1142N, G1292R	1000	108	603

MN: hiPSC derived motor neurons, RSC: primary rat spinal cord cells.

**Table 3:** List of oligonucleotide primers used in this study.

Oligonucleotide	Sequence (5′–3′)	Utility
A1LC-F(Nde)	GCCATATGCCATTTGTTAATAAACAATTTAATTATAAAGATCC	Amplification of *bont*/A1
A1HC-R(Nhe)	GCGCTAGCTTACAGTGGCCTTTCTCCCCATCCATCATCTAC	
A1_GS(1141,2)DN	AGGGCCTAGAGATAACGTAATGACTA	substitutions of G^1141^D and S^1142^N in *bont*/A1
A1_G(1292)R	GTAGATGATGGATGGAGAGAAAGGCCACTG	substitutions of G^1292^R in *bont*/A1
A4_DN(1141,2)GS	AGGGCCTAGAGGTAGCGTAATGACTA	substitutions of D^1141^G and N^1142^S in *bont*/A4
A4_R(1292)G	GATGATGGATGGGGAGAAAGGCCACTGTAAGCTAGC	substitutions of R^1292^G in *bont*/A4
A4-Nde2029	GGTACTTTTGCACTTGTATCTTATGTTTCGAATAAGGTTCTAACCG	removal of internal *Nde*I site in *bont*/A4
A1LC-F	GCCATATGCCATTTGTTAATAAACAATTTAATTATAAAGATCC	Amplification of *bont*/A1 LC with overlap region of *bont*/A4 HC for generation of hybrid toxin gene
A1/4-LCR	CTTTAATACATAACTCATTTAATGCCTTATTATATCC	
A1/4HC-F	CATTAGTAAAATTATCTTCTGAAGGACTAAAAAACAAGTCCC	Amplification of *bont*/A1 HC with overlap region of *bont*/A4 LC for generation of hybrid toxin gene
A1HC-R	GCGCTAGCTTACAGTGGCCTTTCTCCCCATCCATCATCTAC	
A4LC-F	CGCATATGCCATTTGTTAATAAACAATTTAATTATAATGATCCTG	Amplification of *bont*/A4 LC with overlap region of *bont*/A1 HC for generation of hybrid toxin gene
A4/1-LCR	CTTTGATACATAAATCATTTAATGCCTTATTGTATCC	
A4/1-HCF	GGATACAATAAGGCATTAAATGAGTTATGTATCAAAG	Amplification of *bont*/A4 HC with overlap region of *bont*/A1 LC for generation of hybrid toxin gene
A4HC-R	GCGCTAGCTTACAGTGGCCTTTCTCTCCATCCATC	

Underlined restriction sites for *Nde*I, CATATG; *Nhe*I, GCTAGC; LC, light chain; HC, heavy chain.

## Data Availability

The data presented in this study are presented within the article. Original data are available upon request from the corresponding author subject to approval the University of Wisconsin—Madison Biosecurity Task Force.

## Supplementary Materials

The supporting information can be downloaded at https://www.mdpi.com/article/10.3390/ijms24065690/s1. Reference [56] is cited in the supplementary materials.
