# Supplementary figures and images for: Botulinum Neurotoxin A4 Has a 1000-Fold Reduced Potency Due to Three Single Amino Acid Alterations in the Protein Receptor Binding Domain
Source: Int J Mol Sci. 2023 Mar 16;24(6):5690. doi: 10.3390/ijms24065690 (PMC10055998; doi:10.3390/ijms24065690)

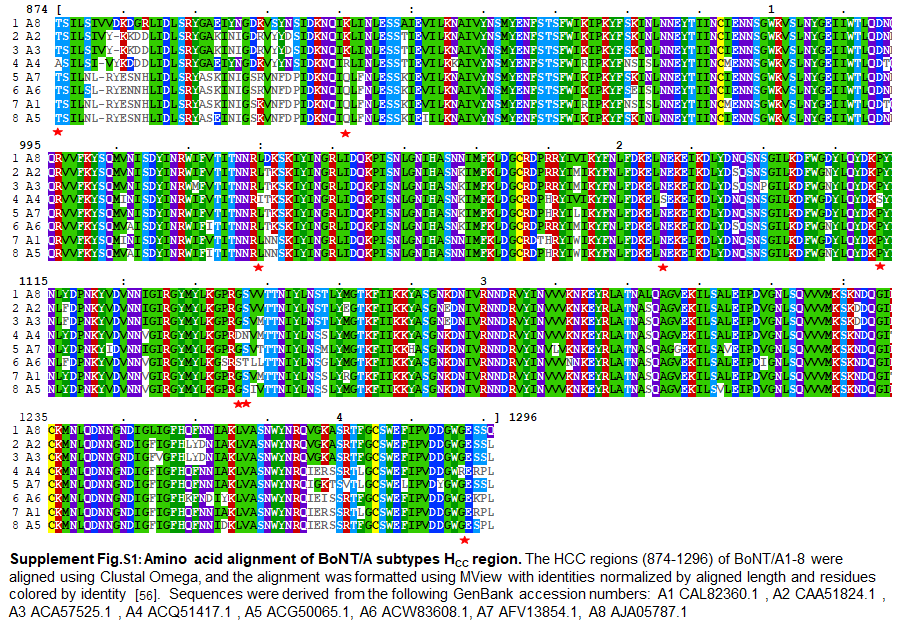

Supplement: Supplementary file 1 [file ijms-24-05690-s001.zip › supplementary Figure S1.tif]

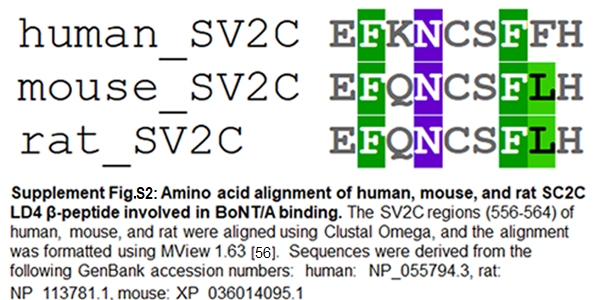

Supplement: Supplementary file 1 [file ijms-24-05690-s001.zip › Supplementary Figure S2.tif]
